# Supplementary material for: Modeling glioblastoma heterogeneity as a dynamic network of cell states
Source: Mol Syst Biol. 2021 Sep 16;17(9):e10105. doi: 10.15252/msb.202010105 (PMC8444284; doi:10.15252/msb.202010105)
Supplement: Supplementary file 6 — Source Data for Figure 5 [file MSB-17-e10105-s004.zip › Figure5A_sourcedata/GSEA_3017/nosigdb_stateE.GseaPreranked.1622795031093/RICHARDS_NOWAKOWSKI_SCIENCE_2017_EN_PFC2_UPREG.html]

Details for gene set RICHARDS\_NOWAKOWSKI\_SCIENCE\_2017\_EN\_PFC2\_UPREG[GSEA]

|  || Dataset | state63017 |
| Phenotype | NoPhenotypeAvailable |
| Upregulated in class | na\_pos |
| GeneSet | RICHARDS\_NOWAKOWSKI\_SCIENCE\_2017\_EN\_PFC2\_UPREG |
| Enrichment Score (ES) | 0.43037736 |
| Normalized Enrichment Score (NES) | 3.3752267 |
| Nominal p-value | 0.0 |
| FDR q-value | 0.0 |
| FWER p-Value | 0.0 |
Table: GSEA Results Summary

  

Fig 1: Enrichment plot: RICHARDS\_NOWAKOWSKI\_SCIENCE\_2017\_EN\_PFC2\_UPREG      
 Profile of the Running ES Score & Positions of GeneSet Members on the Rank Ordered List

  

| PROBE | GENE SYMBOL | GENE\_TITLE | RANK IN GENE LIST | RANK METRIC SCORE | RUNNING ES | CORE ENRICHMENT || 1 | LRRN3 |  |  | 10 | 0.943 | 0.0129 | Yes |
| 2 | RCAN1 |  |  | 17 | 0.750 | 0.0255 | Yes |
| 3 | PRSS23 |  |  | 21 | 0.722 | 0.0409 | Yes |
| 4 | MAP1B |  |  | 22 | 0.715 | 0.0596 | Yes |
| 5 | MSMO1 |  |  | 28 | 0.648 | 0.0707 | Yes |
| 6 | LIMCH1 |  |  | 29 | 0.641 | 0.0875 | Yes |
| 7 | CYP51A1 |  |  | 30 | 0.637 | 0.1042 | Yes |
| 8 | HMGCS1 |  |  | 37 | 0.593 | 0.1127 | Yes |
| 9 | NEDD9 |  |  | 38 | 0.590 | 0.1282 | Yes |
| 10 | KCNJ6 |  |  | 39 | 0.586 | 0.1435 | Yes |
| 11 | MEG3 |  |  | 42 | 0.562 | 0.1559 | Yes |
| 12 | FRMD4B |  |  | 48 | 0.532 | 0.1639 | Yes |
| 13 | CAMK2N1 |  |  | 49 | 0.530 | 0.1778 | Yes |
| 14 | ENC1 |  |  | 58 | 0.501 | 0.1815 | Yes |
| 15 | GAP43 |  |  | 61 | 0.494 | 0.1921 | Yes |
| 16 | PDP1 |  |  | 69 | 0.454 | 0.1958 | Yes |
| 17 | DOK5 |  |  | 81 | 0.437 | 0.1942 | Yes |
| 18 | IRS2 |  |  | 85 | 0.426 | 0.2019 | Yes |
| 19 | VLDLR |  |  | 87 | 0.419 | 0.2117 | Yes |
| 20 | BEX1 |  |  | 88 | 0.419 | 0.2227 | Yes |
| 21 | NDFIP1 |  |  | 91 | 0.416 | 0.2312 | Yes |
| 22 | CHL1 |  |  | 103 | 0.392 | 0.2285 | Yes |
| 23 | ELMOD1 |  |  | 104 | 0.391 | 0.2388 | Yes |
| 24 | RTN4 |  |  | 109 | 0.388 | 0.2442 | Yes |
| 25 | EML1 |  |  | 110 | 0.383 | 0.2543 | Yes |
| 26 | AFF3 |  |  | 111 | 0.382 | 0.2643 | Yes |
| 27 | MAP4 |  |  | 115 | 0.367 | 0.2704 | Yes |
| 28 | LONRF2 |  |  | 118 | 0.365 | 0.2776 | Yes |
| 29 | FAM200B |  |  | 120 | 0.361 | 0.2859 | Yes |
| 30 | GRIA1 |  |  | 122 | 0.359 | 0.2941 | Yes |
| 31 | FHL1 |  |  | 126 | 0.351 | 0.2997 | Yes |
| 32 | SEMA3A |  |  | 128 | 0.349 | 0.3077 | Yes |
| 33 | DSTN |  |  | 129 | 0.348 | 0.3168 | Yes |
| 34 | KHDRBS3 |  |  | 132 | 0.346 | 0.3236 | Yes |
| 35 | SHISA9 |  |  | 133 | 0.345 | 0.3326 | Yes |
| 36 | ARL6IP5 |  |  | 134 | 0.345 | 0.3417 | Yes |
| 37 | NUDT3 |  |  | 136 | 0.343 | 0.3495 | Yes |
| 38 | DAAM1 |  |  | 138 | 0.343 | 0.3573 | Yes |
| 39 | CPEB4 |  |  | 143 | 0.341 | 0.3615 | Yes |
| 40 | NRCAM |  |  | 144 | 0.339 | 0.3704 | Yes |
| 41 | WWC1 |  |  | 154 | 0.329 | 0.3684 | Yes |
| 42 | TUBB2A |  |  | 157 | 0.327 | 0.3746 | Yes |
| 43 | MVD |  |  | 158 | 0.327 | 0.3832 | Yes |
| 44 | DPYSL3 |  |  | 161 | 0.325 | 0.3894 | Yes |
| 45 | DCBLD2 |  |  | 165 | 0.322 | 0.3943 | Yes |
| 46 | SQLE |  |  | 168 | 0.319 | 0.4003 | Yes |
| 47 | NFIX |  |  | 171 | 0.317 | 0.4062 | Yes |
| 48 | TNIK |  |  | 178 | 0.312 | 0.4073 | Yes |
| 49 | GAS7 |  |  | 180 | 0.310 | 0.4143 | Yes |
| 50 | SPTBN1 |  |  | 194 | 0.300 | 0.4068 | Yes |
| 51 | KIFAP3 |  |  | 208 | 0.290 | 0.3990 | Yes |
| 52 | NLN |  |  | 214 | 0.286 | 0.4007 | Yes |
| 53 | IDH1 |  |  | 218 | 0.284 | 0.4046 | Yes |
| 54 | SCG2 |  |  | 220 | 0.283 | 0.4108 | Yes |
| 55 | SRGAP1 |  |  | 223 | 0.281 | 0.4158 | Yes |
| 56 | CXADR |  |  | 232 | 0.275 | 0.4136 | Yes |
| 57 | IAH1 |  |  | 244 | 0.265 | 0.4075 | Yes |
| 58 | CSRP2 |  |  | 247 | 0.264 | 0.4121 | Yes |
| 59 | HMGCR |  |  | 251 | 0.261 | 0.4154 | Yes |
| 60 | INSIG1 |  |  | 252 | 0.261 | 0.4222 | Yes |
| 61 | PGM2L1 |  |  | 255 | 0.260 | 0.4267 | Yes |
| 62 | ATP9A |  |  | 264 | 0.255 | 0.4239 | Yes |
| 63 | FDFT1 |  |  | 266 | 0.252 | 0.4294 | Yes |
| 64 | MICU1 |  |  | 279 | -0.253 | 0.4218 | Yes |
| 65 | TUBA1C |  |  | 282 | -0.253 | 0.4261 | Yes |
| 66 | DRAXIN |  |  | 285 | -0.254 | 0.4304 | Yes |
| 67 | FAT3 |  |  | 311 | -0.261 | 0.4077 | No |
| 68 | HNRNPAB |  |  | 312 | -0.261 | 0.4146 | No |
| 69 | CPE |  |  | 315 | -0.262 | 0.4191 | No |
| 70 | RAD51C |  |  | 333 | -0.264 | 0.4059 | No |
| 71 | SLK |  |  | 343 | -0.266 | 0.4023 | No |
| 72 | EPB41 |  |  | 345 | -0.268 | 0.4081 | No |
| 73 | ADCY1 |  |  | 347 | -0.268 | 0.4140 | No |
| 74 | HDAC2 |  |  | 348 | -0.268 | 0.4210 | No |
| 75 | PGD |  |  | 355 | -0.270 | 0.4210 | No |
| 76 | CERS6 |  |  | 375 | -0.275 | 0.4058 | No |
| 77 | RNF165 |  |  | 382 | -0.276 | 0.4060 | No |
| 78 | SLC12A2 |  |  | 389 | -0.279 | 0.4062 | No |
| 79 | CDC123 |  |  | 394 | -0.280 | 0.4088 | No |
| 80 | GDI2 |  |  | 401 | -0.283 | 0.4092 | No |
| 81 | SLIT1 |  |  | 408 | -0.285 | 0.4095 | No |
| 82 | PPA1 |  |  | 420 | -0.289 | 0.4041 | No |
| 83 | SATB2 |  |  | 429 | -0.292 | 0.4023 | No |
| 84 | TTL |  |  | 440 | -0.297 | 0.3983 | No |
| 85 | HOMER1 |  |  | 463 | -0.304 | 0.3803 | No |
| 86 | PA2G4 |  |  | 501 | -0.314 | 0.3449 | No |
| 87 | TSPAN14 |  |  | 581 | -0.350 | 0.2608 | No |
| 88 | MTURN |  |  | 583 | -0.350 | 0.2688 | No |
| 89 | ASRGL1 |  |  | 608 | -0.362 | 0.2499 | No |
| 90 | CDKN2D |  |  | 638 | -0.381 | 0.2257 | No |
| 91 | PID1 |  |  | 645 | -0.385 | 0.2287 | No |
| 92 | RNF157 |  |  | 669 | -0.409 | 0.2123 | No |
| 93 | SOGA1 |  |  | 672 | -0.411 | 0.2207 | No |
| 94 | GNG4 |  |  | 676 | -0.415 | 0.2280 | No |
| 95 | PCDH10 |  |  | 757 | -0.491 | 0.1464 | No |
| 96 | DCX |  |  | 769 | -0.513 | 0.1469 | No |
| 97 | CMC2 |  |  | 771 | -0.518 | 0.1593 | No |
| 98 | STMN1 |  |  | 778 | -0.529 | 0.1661 | No |
| 99 | TUBB |  |  | 802 | -0.570 | 0.1539 | No |
| 100 | CEP78 |  |  | 803 | -0.570 | 0.1688 | No |
Table: GSEA details [plain text format]

  

Fig 2: RICHARDS\_NOWAKOWSKI\_SCIENCE\_2017\_EN\_PFC2\_UPREG: Random ES distribution      
 Gene set null distribution of ES for **RICHARDS\_NOWAKOWSKI\_SCIENCE\_2017\_EN\_PFC2\_UPREG**
